# CTGF as a multifunctional molecule for cartilage and a potential drug for osteoarthritis

**DOI:** 10.3389/fendo.2022.1040526

**Published:** 2022-10-17

**Authors:** Zihuan Yang, Weishi Li, Chunli Song, Huijie Leng

**Affiliations:** ^1^ Department of Orthopaedics, Peking University Third Hospital, Beijing, China; ^2^ Engineering Research Center of Bone and Joint Precision Medicine, Beijing, China; ^3^ Beijing Key Laboratory of Spinal Disease Research, Beijing Municipal Science & Technology Commission, Beijing, China

**Keywords:** CTGF, chondrocyte, cartilage, degeneration, osteoarthritis treatment

## Abstract

CTGF is a multifunctional protein and plays different roles in different cells and under different conditions. Pamrevlumab, a monoclonal antibody against CTGF, is an FDA approved drug for idiopathic pulmonary fibrosis (IPF) and Duchenne muscular dystrophy (DMD). Recent studies have shown that CTGF antibodies may potentially serve as a new drug for osteoarthritis (OA). Expression of CTGF is significantly higher in OA joints than in healthy counterparts. Increasing attention has been attracted due to its interesting roles in joint homeostasis. Joint homeostasis relies on normal cellular functions and cell-cell interactions. CTGF is essential for physiological activities of chondrocytes. Abnormal CTGF expression may cause cartilage degeneration. In this review, the physiological functions of CTGF in chondrocytes and related mechanisms are summarized. Changes in the related signaling pathways due to abnormal CTGF are discussed, which are contributing factors to inflammation, cartilage degeneration and synovial fibrosis in OA. The possibility of CTGF as a potential therapeutic target for OA treatment are reviewed.

## Introduction

Connective tissue growth factor (CTGF), also named as HCS24, Ecogenin, Regenerin and CCN2, is a member of the CCN family (CTGF, Cyr61, NOV). CTGF is a multifunctional secretory protein in a variety of fibrous tissues, and involved in numerous physiological and pathophysiological processes including embryonic development, tumor formation and osteoarthritis (OA) progression (1).

CTGF, 38 kDa, consists of 4 domains, including insulin-like growth factor binding protein domains (IGFBP), von Willbrand factor C-type repeat domains (VWC), thrombospondin type-1 repeat domains (TSP-1) and cysteine knot-containing domains (CT) (2). IGFBP and VWC constitute the N-terminus of CTGF. TSP-1 and CT constitute the C-terminus of CTGF. Non-conserved protease-regulating hinge connects the N-terminal and C-terminal. All the domains have distinctive functions (3). The distictive structure provides CTGF the capability to participate in various biological processes (4).

OA is a common joint disease, mainly featured as cartilage degeneration, subchondral sclerosis and synovial inflammation (5). CTGF is involved in chondrogenesis and OA processes (6). The roles of CTGF in OA are complicated (7–9). This review summarizes the current understanding of the physiological roles of CTGF in chondrocytes and the related mechanisms for OA. The potential of CTGF as a therapeutic target for OA treatment is also briefly reviewed.

## Under the physiological condition

Under the normal physiological condition, CTGF can mediate chondrocyte activities and cartilage matrix synthesis, sense environmental stimulation, and promote celluar communication. The roles and related mechanisms of CTGF in chondrocytes under the normal physiological condition better illustrate its multifunctional attributes.

### Involvement in chondrocyte activities

CTGF can boost chondrocyte proliferation. Overexpression of *Ctgf* or treatment with recombinant CTGF (rCTGF) leads to increased proliferation of HCS-2/8 chondrocytes (10, 11). The similar results were observed in rabbit chondrocytes (12). Proliferation cell nuclear antigen (PCNA) is closely related to DNA synthesis and is a specific proliferation marker (13). A large number of PCNA positive chondrocytes were observed in the epiphyseal proliferation zone of *Col2a1-ccn2* transgenic mice (14). CTGF was considered to regulate chondrocyte proliferation *via* MAPK and PI3K/Akt signaling pathways (15–18). The main branches of the MAPK signaling pathway include the JNK, ERK and p38MAPK pathways (19). CTGF stimulates proliferation of HCS-2/8 chondrocytes and rabbit growth plate chondrocytes through ERK (15, 16). CTGF was reported to activate the ERK signaling pathway through PKC and MEK1/2 (16). On the other hand, CTGF can form complexes by combining with cytokines directly, such as BMP2 and FGF2 (20–22). It is worth noting that responses of chondrocytes change with different complexes with FGF2. The heterocomplex formed by CTGF and FGF2 can promote proliferation of chondrocytes due to the ability to interact with FGFR1. However, the heterocomplex formed by CT module and FGF2 inhibits proliferation of chondrocytes possibly due to the inability of the CT module to bind to FGFR1 (22).

CTGF participates in several essential processes including mesenchymal cell (MSC) aggregation and terminal differentiation. *In vitro* culture of C3H10T1/2 cells showed that CTGF could induce MSC aggregation and upregulate expression of *Sox9*, a marker of chondrogenic differentiation (23). The roles of CTGF in chondrocyte differentiation depend on the specific types of cells. Under stimulation with rCTGF, COL-X and ALP, the two markers of terminal differentiation, were detected in the growth plate chondrocytes, but not in the articular chondrocytes (11). Hypertrophic differentiation of chondrocytes is highly related with endochondral osteogenesis. CTGF was proven to be an “ecogenin” (endochondral ossification genetic factor) (1, 10, 11). The expression of ALP can be increased by CTGF through p38MAPK pathway and PI3K pathway, thereby promoting endochondral ossification (15).

### Sensing environmental stimulation

Chondrocytes are regulated by mechanical stimuli, which are eventually converted into biological effects by inhibiting or activating signaling pathways (24). Abnormal mechanical loading causes imbalance of chondrocyte homeostasis and promotes the occurrence and development of OA 25. CTGF is one of the “mechanical stimulation receptors” of chondrocytes (7). Significant differences were observed in the distribution and expression of CTGF at different stages of OA and in different regions of cartilage which reflected different loading conditions (26). The expression of CTGF in articular cartilage was inhibited after 4 h of cyclic loading 27. Tensile stress can induce significantly higher expression of CTGF than hydrostatic pressure (28). Different intensities of pulsed ultrasound have various effects on the production of CTGF (29).

In chondrocytes, CTGF not only senses mechanical stimulation, but also participates in transferring mechanical signals into biochemical signals in chondrocytes. CTGF transmits mechanical signals through molecules which are distributed in the cell membrane, thereby activating or inhibiting downstream signaling pathways (30–32). CTGF, as a ligand of integrins, also regulates cellular activities induced by mechanical loading through integrin pathways (7). Another way for CTGF to convert mechanical cues into biochemical signals is to activate Smad2/3 signaling in articular cartilage (33).

Expression of CTGF in chondrocytes demonstrates rhythmic changes, possibly responsible for the circadian rhythm of chondrocytes (34). During the diurnal cycle, cartilage and chondrocytes experience cyclic high- and low-intensity loadings, which is reflected by the diurnal variation in cartilage thickness at the distal femur 35. Rhythmic changes were also observed in pain and in the expressions of chondrocyte-related markers such as ACAN, COL-II, COMP (36). Proteomic sequencing of mouse articular cartilage showed that, among the 146 proteins, only expression of CTGF fluctuated with a cycle of 16 hours (34).

### Mediating cartilage matrix synthesis

Cartilage is in a dynamic balance between extracellular matrix (ECM) synthesis and decomposition. Under the normal physiological condition, CTGF is a matrix protein involved in extracellular matrix protein synthesis (37). By knocking out *Ctgf* in chondrocytes, synthesis of COL-II and proteoglycan (PG) in ECM was reduced (10). Several researches reported that CTGF can combine with different molecules to directly or indirectly regulate synthesis and degradation of cartilage ECM. The CTGF and MMP family can synergistically function in cartilage ECM (38). Cotransfection of *Ctgf* and *Timp-1* (MMP-specific inhibitory gene) can significantly promote the expression of PG and COL-II (39). By cutting the hinge between VWC and TSP1 in CTGF, MMPs release C-terminal fragments, which can activate Akt and ERK pathways (40, 41). CTGF, the downstream of the TGF-β signaling pathway, can also interact with the TGF-β family to influence cartilage ECM synthesis (42).

CTGF can regulate ECM formation by directly binding to ECM proteins or through cellular energy metabolism. Destruction of the interaction between CTGF and ACAN can lead to inhibition of chondrogenesis (43). The IGFBP-VWC region of CTGF promotes the synthesis and deposition of ACAN by connecting to the ACAN G3 domain (44). However, binding with the CT domain might be independent of ECM protein synthesis (45). ECM protein synthesis requires energy consumption. Adenosine triphosphate, also known as ATP, is a characteristic molecule representing energy in cells. Since cartilage is an avascular tissue, its main type of metabolism is glycolysis (46). ATP in chondrocytes can be effected by glycolysis and availability of free amino acids. In *Ctgf-*null chondrocytes, the free amino acid can not be captured by CTGF and glycolysis is impared, causing ATP shortage and energy production decrease (47, 48). Metabolomics and transcriptome sequencing of *Ctgf*-null chondrocytes also revealed low levels of ATP, ENO1 (one of the glycolysis enzymes) and ACAN (47).

### Regulating communication among cells

CTGF is also known as cellular communication network factor 2 (CCN2), which acts as a signal conductor for communication among chondrocytes by intercellular junctions between chondrocytes (49). Under rCTGF intervention, the migration efficiency of chondrocytes increased in a concentration-dependent manner, and the formation of intercellular junctions of chondrocytes were significantly increased. Cx43, as the main connexin of chondrocytes, is one of the semichannel structures of gap junctions (50). CTGF can induce Cx43 opening to promote the exchange of miRNA, ATP and Ga^+^, through the PI3K/Akt signaling pathway (49).

CTGF can also mediate communication between chondrocytes and other types of cells, for example, osteoclast, osteoblast, and endotheliocyte (1, 51, 52). Increased secretion of CTGF in OUMS-27 cells (a human chondrocyte cell line) due to TNF-α treatment has a synergistic effect on osteoclastogenesis in coordination with M-CSF/RANKL. The possible mechanism was proposed that CTGF from chondrocytes can activate FAK and ERK1/2 through binding to αVβ3 on osteoclasts (53). CTGF secreted by chondrocytes can also act on osteoblasts close to the hypertrophic zone of cartilage in a paracrine manner during endochondral osteogenesis (54).

## Under the OA condition

**Figure 1:**
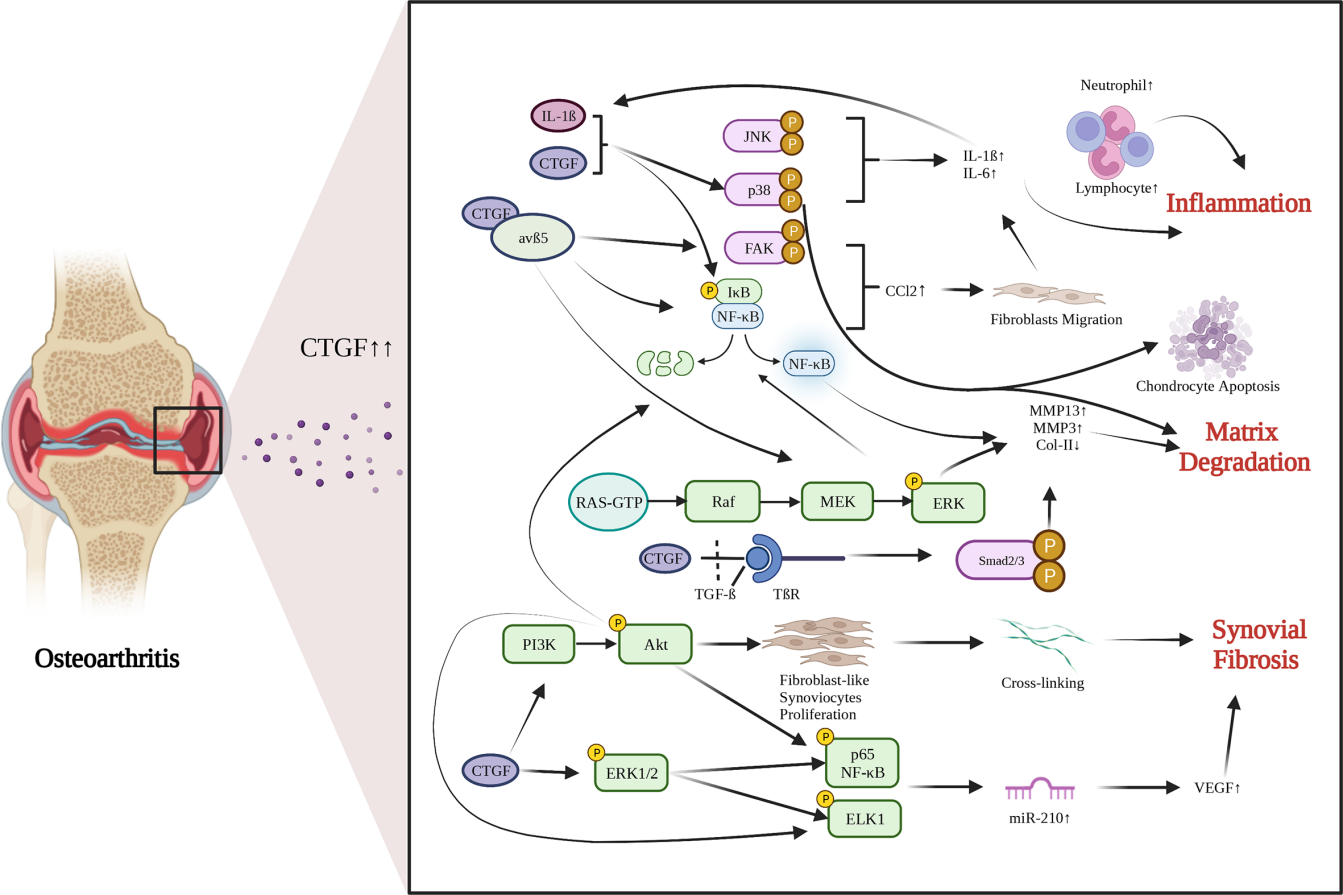
Functions and pathways of CTGF in OA development. CTGF is involved in promoting inflammation, matrix degradation and synovial fibrosis in articular joints. Created with biorender.com.

OA is a common chondro-related degenerative disease. Chondrocytes are the main cell in cartilage. Phenotypic changes of chondrocytes are the important characteristics of OA. CTGF is gradually recognized as an important mediator of chondrocyte activities. There are growing interests in identifying the role of CTGF in OA recently. Several studies have reported an abnormal increase in CTGF expression in OA cartilage (55) and OA synovium (6). The role of CTGF in OA is still in debate. The functions and pathways of CTGF in OA development are summarized in **Figure 1**.

### Cartilage degeneration

As mentioned above, CTGF mediates cartilage ECM synthesis through multiple pathways. However, whether CTGF can repair or aggravate cartilage degeneration is still unclear. The different opinions were based on *in vivo* studies and their designs are summarized in **Table 1**. The inconsistent conclusions might result from differences in types of techniques for CTGF deletion, OA models, animal species/ages/genders, experimental schedules, and OA scoring systems used in those studies. CTGF are widely distributed in other joint tissues such as synovium and bone (63). Selective deletion of CTGF might have different effects from global deletion (56). Different types of OA models represent different pathologies which might affect the microenvironment of CTGF. Proliferative activity of chondrocyte varies with different stages and status (64, 65). Thus, the ages of animal and the schedule of intervention may influence how CTGF functions. CTGF might be sensitive to microenvironment determined by multiple factors.

**Table 1:** Different effects of CTGF on cartilage degeneration *in vivo*.

OA model	Gender	Suppress-/promote+ CTGF Expression	Experimental Schedule	Scoring System	Effect of CTGF on cartilage	Reference
Non-invasive PTOA model/mice	Male	–	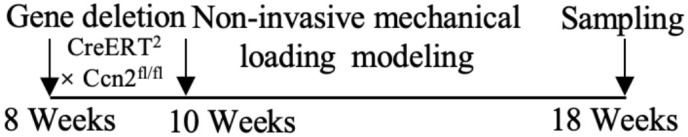	Lesion severity	Not Significant	(56)
DMM-induced OA model/mice	Male	–	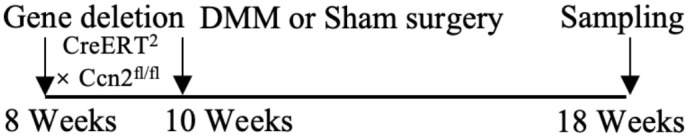	Lesion severity	Not Significant	(56)
DMM-induced OA model/mice	Male/female	–	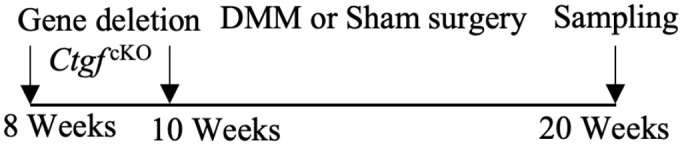	OARSI score	Negative	(57)
Collagenase-induced OA model/mice	Male	–	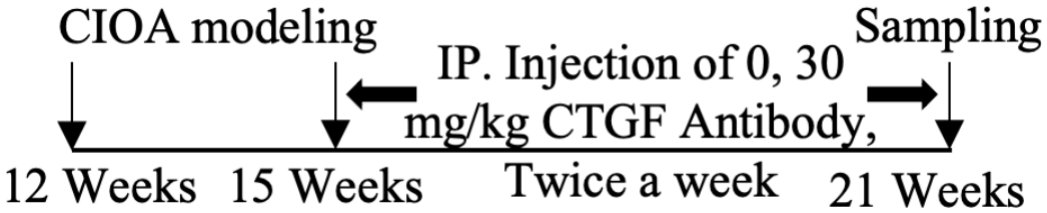	Erosion severity	Negative	(58)
Collagenase-induced OA model/rat	Female	–	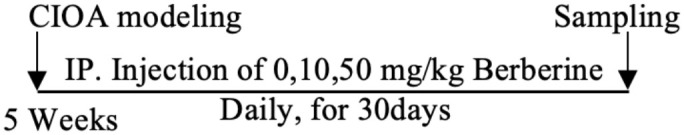	Staining intensity	Negative	(59)
Surgery-induced cartilage defect OA model/rat	Male	+	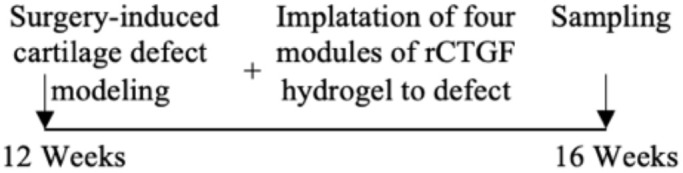	Lesion severity	Positive	(60)
MIA-induced OA model/rat	Male	+	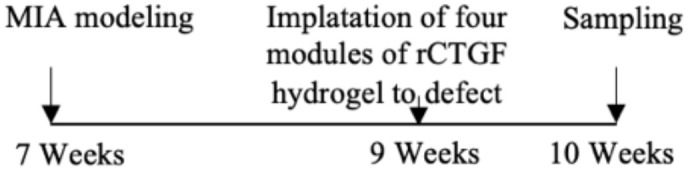	Lesion severity	Positive	(60)
MIA-induced OA model/rat	Male	+	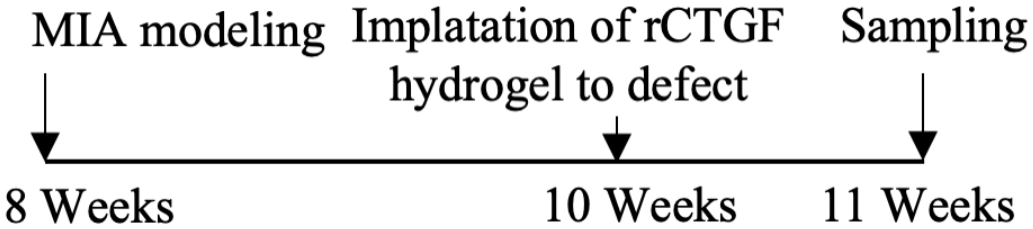	Wakitani Scores	Positive	(61)
Age-related OA model/mice	Male	+	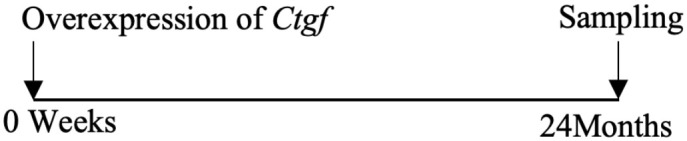	Staining intensity	Positive	(62)

CTGF is essentially a multifunctional molecule that can combine with different cytokines and receptors. CTGF can combine with TGF-β to form a complex through disulfide bonds in chondrocytes (57). After abnormal mechanical stimulation, the complex binds to the TGF-β receptor, activates Smad2/3, and releases CTGF from the complex. When *Ctgf* is specifically knocked out, the progression of OA will be inhibited. Tang et al. mentioned that CTGF release may be related to sodium ions, which was proven in Keppies experiment (66). CTGF also controls ECM synthesis by interacting with other molecules, such as Rab14 (67) and Wif-1 (68).

### Synovial fibrosis

Synovial fibrosis is one of the characteristics of OA. CTGF is a profibrotic protein, and is considered to be the main driver of many fibrotic diseases (69). Liao have proposed that CTGF is a highly discriminatory biomarker of synovial fibrosis in OA (70). Adenovirus-*Ctgf* was transfected into the mouse knee synovium, which exacerbated synovial fibrosis (39). Currently, the regulatory mechanisms by which CTGF induces synovial fibrosis in OA remain largely unrevealed. Remst found that TGF-β induces Lysyl hydroxylase 2b in fibroblasts *via* ALK5 signaling (71). CTGF may associated with TGF-β-mediated synovial fibrosis (70). CTGF can induce vascular endothelial growth factor (VEGF) production and angiogenesis in synovial fibroblasts by raising miR-210 expression *via* PI3K, AKT, ERK and NF-κB/ELK1 pathways, leading to aggravated synovial fibrosis (72).

### Inflammation

OA has been considered a low-grade inflammatory disease. Synovial inflammation is realized to play an important role in the development of OA. CTGF, as an inflammatory mediator, is involved in synovial inflammation, inducing synovial cells to produce inflammatory cytokines (73). After treating synovial cells with 0.1 mg/mL sodium hyaluronate, the expression of CTGF was significantly decreased, and the degradation of cartilage was significantly inhibited (74). Monocyte migration can also stimulate production of inflammatory factors. CTGF can induce monocyte migration in OA by activating the downstream FAK, MEK, ERK, and NF-κB signaling pathways through interaction with integrin avβ5, and by enhancing generation and activation of CCL2, a promotor for monocyte migration (75). When synovial fibroblasts are treated with rCTGF, CTGF and integrin avβ5 jointly activate the p38 MAPK and JNK pathways, and increase the entry of p65 NF-κB and C-Jun into the nucleus to induce IL-6 expression (76). CTGF can also activate the ERK1/2, p38 MAPK and p65 NF-κB signaling pathways and increase the production of inflammatory factors and chondrocyte catabolic markers through synergistic interaction with IL-1β (77). These findings imply that CTGF acts as an inducer of inflammatory cytokines and promoter of inflammation in OA.

## Current therapeutic strategy targeting CTGF

CTGF plays a critical role in the physiological and pathological states of cartilage, indicating it may be a therapeutic target for OA. Although some scholars have considered rCTGF as a cartilage regeneration strategy (78, 79), a large amount of recent data have proven that suppressing CTGF expression and antagonizing CTGF activities are effective in combatting OA. Antibodies, natural compounds, gene knockout and other interventions were used to target CTGF for OA treatment.

At the protein level, the therapeutic effects of targeting CTGF are most focused on CTGF antibody. Pamrevluma (FG-3019), a monoclonal antibody against CTGF, has already been in phase II clinical trials for treatment of PIF and DMD (80). Its application to OA treatment has not yet been reported. Miniato developed CTGF antibodies against four different modules of CTGF. Among them, IGFBP, VWC and CT module-specific antibodies can promote PG synthesis (81). Other inhibitors of OA that target CTGF include sodium hyaluronate (73), PGE2 (Prostaglandin E2); (82), AP-1 inhibitor (Curcumin), IL-1β inhibitor Berberine (59), CTGF upstream inhibitor ROCK inhibitor (Y27632) and CTGF downstream inhibitors, such as avβ5 integrin-neutralizing antibody and NF-κB inhibitor IKK (76). Berberine, is the main anti-inflammatory component of the Chinese herb Rhizoma coptidis (Huanglian). Thirty-day injection of Berberine was able to inhibit the expression of IL-1β and cartilage degeneration induced by the collagenase (59).

Great advancements in gene therapy for OA has been achieved through preclinical and clinical studies (83). Target CTGF for OA treatment at the gene level were also investigated recently. Caos study showed that *miR-296-5p* could inhibit the abnormal increase in CTGF expression, preventing chondrocyte apoptosis and cartilage matrix degradation in IL-1β induced OA (77). LncRNA *Pvt1* knockdown can effectively improve collagen degradation caused by abnormally elevated CTGF in diabetes-mediated OA (84). Knocking out *Ctgf* by genome editing technology can significantly inhibit the OA process (57). Gene therapy targeting CTGF will be a promising strategy for OA.

## Conclusion

CTGF is a multifunctional molecule that has great potential as a diagnostic marker and therapeutic target in OA. As an inflammatory factor, although CTGF plays an irreplaceable role in promoting chondrogenesis, it also serves as a driving factor for pathological changes in OA. It is far from clear why CTGF functions differently and how it works. Other than exploring the complex signal transduction and the different microenvironment, investigating different binding targets and different distribution of binding sites probably can explain varying effects of CTGF on chondrocytes, and shed more lights on how can we target CTGF as a treatment of OA.

## Author contributions

ZY took responsibility for drafting the manuscript. ZY, WL, and CS participated in the collection, sorting, and analysis of documents. HL gave critical direction on the review and revised the manuscript. All authors contributed to the article and approved the submitted version.

## Funding

This work was supported by the National Natural Science Foundation of China (Grant number: 12172011, 11872076, 11472017).

## Conflict of interest

The authors declare that the research was conducted in the absence of any commercial or financial relationships that could be construed as a potential conflict of interest.

## Publisher’s note

All claims expressed in this article are solely those of the authors and do not necessarily represent those of their affiliated organizations, or those of the publisher, the editors and the reviewers. Any product that may be evaluated in this article, or claim that may be made by its manufacturer, is not guaranteed or endorsed by the publisher.
